# Influence of reminiscence therapy on mental health and quality of life in elderly patients with unresectable, metastatic gastrointestinal cancer

**DOI:** 10.1590/1414-431X2024e13344

**Published:** 2024-05-20

**Authors:** Yu Liang, Limin Zhang

**Affiliations:** 1 Department of Gastrointestinal Surgery, Cancer Hospital, Harbin Medical University, Harbin, China

**Keywords:** Elderly patients with metastatic gastrointestinal cancer, Reminiscence therapy, Anxiety and depression, Spiritual well-being, Quality of life

## Abstract

Reminiscence therapy (RT) attenuates psychological disorders in cancer patients. This study aimed to evaluate the effect of RT on anxiety, depression, spiritual well-being, and quality of life in elderly patients with unresectable, metastatic gastrointestinal cancer. A total of 222 elderly patients with unresectable, metastatic gastrointestinal cancer were randomized into RT group (RT plus usual care, n=112) or control group (usual care, n=110) with a 6-month intervention. Hospital Anxiety and Depression Scale for Anxiety (HADS-A) and Depression (HADS-D), Functional Assessment of Chronic Illness Therapy-Spiritual Well-Being Scale (FACIT-Sp), and Quality of Life Questionnaire-Core 30 (QLQ-C30) were evaluated at month (M)0, M1, M3, and M6. Concerning the primary outcome, HADS-A score at M6 decreased in the RT group compared to the control group (P=0.005). As to secondary outcomes, the RT group showed decreased HADS-A scores at M3, anxiety rate at M3, HADS-D scores at M3 and M6, depression rate at M6, as well as greater FACIT-Sp scores at M1, M3, and M6 *vs* the control group (all P<0.050). Additionally, QLQ-C30 global health score was elevated at M1 (P=0.046) and M6 (P=0.005), functions score was greater at M6 (P=0.038), and symptoms score was lower at M3 (P=0.019) in the RT group than in the control group. Subgroup analysis revealed that the addition of RT was more effective for patients with anxiety or depression at baseline. In summary, RT alleviated anxiety and depression, and improved the spiritual well-being and quality of life within 6 months in elderly patients with unresectable, metastatic gastrointestinal cancer.

## Introduction

Gastrointestinal (GI) cancer, comprising colorectal cancer, gastric cancer, liver cancer, esophageal cancer, and pancreatic cancer, accounts for more than one-quarter of cancer cases and more than one-third of cancer-related deaths worldwide (1). Generally, a proportion of GI cancer patients experiencing cancer metastasis have relatively low surgical feasibility, which may result in anxiety, depression, worse spiritual well-being, and an unsatisfying quality of life (QoL) (2-4). Among them, elderly patients are potentially more vulnerable to these psychological disorders and poor QoL (5). As a result, seeking effective nursing interventions to relieve psychological disorders and improve spiritual well-being and QoL is crucial for the clinical management of elderly patients with unresectable, metastatic GI cancer.

Reminiscence therapy (RT) is a nursing intervention used to attenuate mental disorders (including anxiety and depression) by sharing indelible stories, best-loved things, etc. (6-8). Recent studies suggest that RT is effective at relieving anxiety and depression and improving QoL in GI cancer patients (9,10). For instance, one previous study reported that telephone-based RT reduces the symptoms of depression in colorectal cancer patients who undergo postoperative chemotherapy (10). Another study suggested that RT decreases the anxiety rate and raises the QoL in postoperative gastric cancer patients (9). However, the application of RT in elderly patients with unresectable, metastatic GI cancer is scarce.

Therefore, this randomized controlled study aimed to investigate the effect of RT on anxiety, depression, spiritual well-being, and QoL in elderly patients with unresectable, metastatic GI cancer.

## Material and Methods

### Patients

Between January 2020 and March 2023, 222 elderly patients with unresectable, metastatic GI cancer were enrolled in this randomized, controlled study. Patients who met the following criteria were eligible for inclusion: a) had a diagnosis of gastric cancer or colorectal cancer; b) were aged ≥60 years; c) were able to independently complete the evaluation; d) were capable of and willing to communicate with others; and e) were willing to follow the study protocol. Patients who met the following conditions were excluded: a) had other malignant diseases or b) had severe neurological disease or cognitive dysfunction. The study was approved by the Ethics Committee of the Cancer Hospital, Harbin Medical University. Patients provided written informed consent.

### Randomization

After inclusion, randomization (1:1 ratio) was carried out using the block randomization method (a block size of 4), and the patients were assigned to receive usual care (control group) or RT plus usual care (RT group). In brief, each patient's random assignment information was sealed in an opaque wrapper corresponding to the patient's registration series number.

### Treatment

The interventions were conducted at the rehabilitation center in groups once every two weeks for 6 months after enrollment by two trained nurses. Each session lasted 120 min.

Patients in the control group received usual care, which included health education, follow-up, and frequently asked questions (FAQs). The health education session included an introduction to GI cancer, treatments, management of adverse events, self-monitoring precautions, management of healthy lifestyles, and mental health.

Patients in the RT group received RT (90 min) plus usual care (30 min), and the usual care was the same as that in the control group. The RT consisted of 12 topics, and one topic was chosen for each session: a) an introduction of self and family; b) sharing indelible childhood stories; c) sharing indelible school stories; d) sharing indelible love stories; e) sharing indelible travel stories; f) sharing best-loved movies or songs; g) sharing best-loved books; h) sharing best-loved sports; i) sharing most adored historical figures; j) sharing special skills; k) sharing one of the most memorable items; and l) reviewing and summarizing.

### Assessment

At baseline (M0), 1st month (M1), 3rd month (M3), and 6th month (M6), the Hospital Anxiety and Depression Scales-Anxiety (HADS-A), the Hospital Anxiety and Depression Scales-Depression (HADS-D), the Functional Assessment of Chronic Illness Therapy-Spiritual Well-Being Scale (FACIT-Sp), and the QLQ-C30 scores were evaluated (11-13). Anxiety and depression were considered if the HADS-A or HADS-D score was >7. QLQ-C30 scores included global health status, functions, and symptoms scores. The primary outcome was HADS-A at M6. The secondary outcomes were HADS-A score at M0, M1, and M3, as well as HADS-D, FACIT-Sp, and QLQ-C30 scores at M0, M1, M3, and M6.

### Sample size calculation

Based on clinical experience, we hypothesized that the mean HADS-A score at M6 would be 7 (standard deviation (SD) = 2) in the control group and 6 (SD=2) in the RT group. The minimum sample size was calculated as 85 for each group, with a significance level of 0.05 and a power of 0.90. Given the expected 20% loss to follow-up, a sample size of more than 106 was required for each group.

### Statistics

The analyses were conducted with SPSS v22.0 (IBM, USA) according to the intention-to-treat (ITT) principle. The figures were generated with GraphPad Prism v9.0 (GraphPad Software, Inc., USA). Comparisons were done by Student's *t*-test, chi-squared test, or Wilcoxon rank-sum test to explore the effect of RT on anxiety, depression, spiritual well-being, and quality of life in patients. Subgroup analysis of HADS-A, HADS-D, FACIT-Sp, and QLQ-C30 scores at M6 between the control group and RT group was done using Student's *t*-test. Multivariate linear regression model for HADS-A at M6 was done with the stepwise method. P values <0.05 were considered significant.

## Results

### Study flow

**Figure 1:**
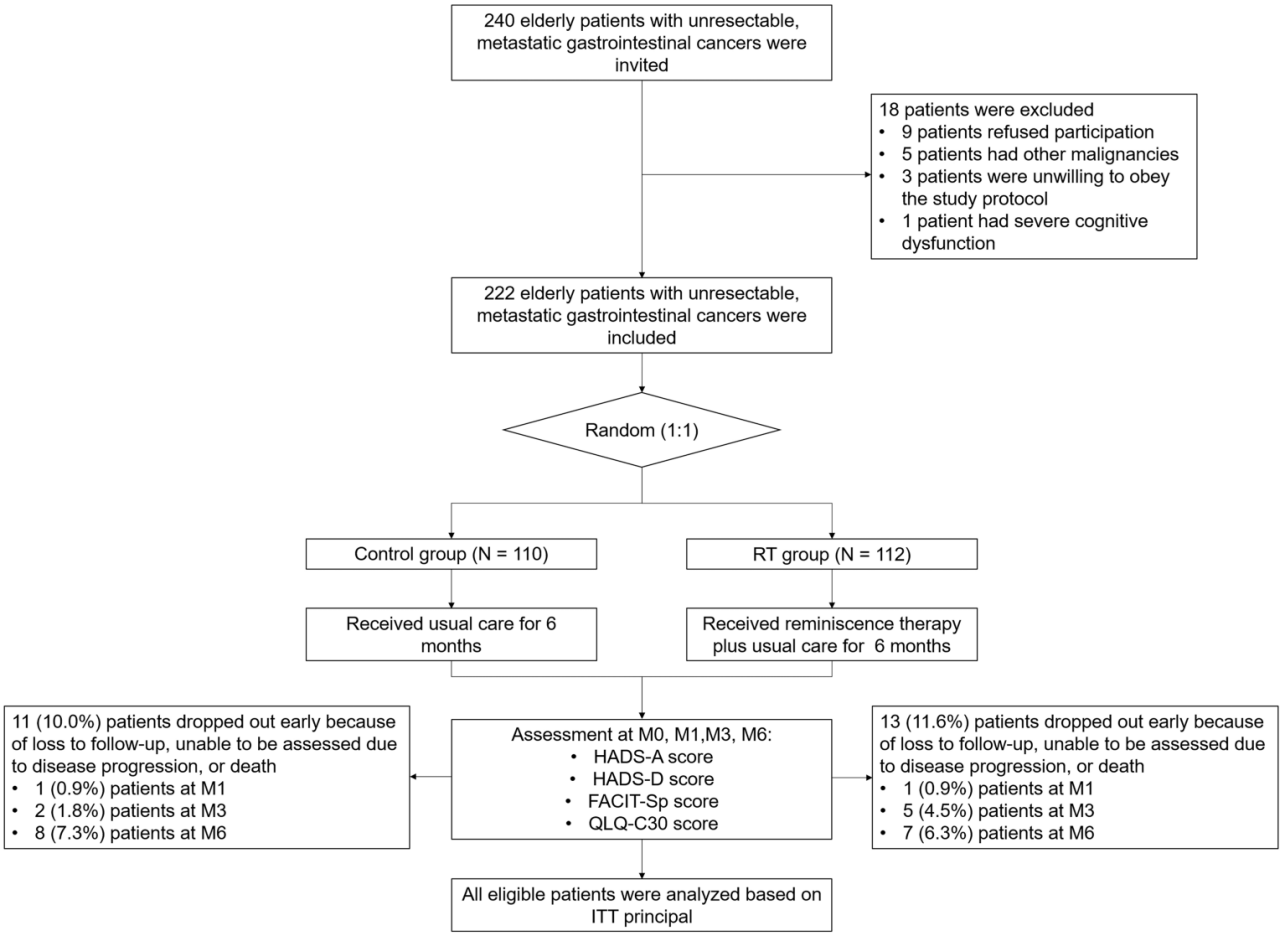
Study flow. RT: Reminiscence therapy; HADS-A: Hospital Anxiety and Depression Scales-Anxiety; HADS-D: Hospital Anxiety and Depression Scales-Depression; FACIT-Sp: Functional Assessment of Chronic Illness Therapy-Spiritual Well-Being Scale; QLQ-C30: Quality of Life Questionnaire-Core 30; M: months; ITT: intention to treat.

Two hundred and forty elderly patients with unresectable metastatic GI cancer were invited to participate in the study; 18 patients were excluded, including 9 patients who refused to participate, 5 patients who had other malignancies, 3 patients who were unwilling to follow the study protocol, and 1 patient who had severe cognitive dysfunction. The remaining 222 patients were then randomized (1:1) into the RT group (n=112) to receive a 6-month RT plus usual care intervention and into the control group (n=110) to receive a 6-month usual care intervention. During the 6-month follow-up period, 13 (11.6%) patients in the RT group and 11 (10.0%) patients in the control group dropped out of this study. The HADS-A, HADS-D, FACIT-Sp, and QLQ-C30 scores were assessed for all patients at M0, M1, M3, and M6. All eligible patients were analyzed based on the ITT principle (Figure 1).

### Clinical characteristics

The mean ages were 68.9±5.5 years and 70.0±6.2 years in the RT and control groups, respectively. In the RT group, there were 38 (33.9%) females and 74 (66.1%) males, and in the control group, there were 32 (29.1%) females and 78 (70.9%) males. Demographic information, medical history, disease information, treatment information, and assessment at M0 were not significantly different between the two groups (all P>0.050). The specific characteristics are reported in Table 1.

**Table 1:** Clinical characteristics of elderly patients with unresectable, metastatic gastrointestinal cancer treated with regular therapy (control) or with regular therapy + reminiscence therapy (RT).

Items	Control group (n=110)	RT group (n=112)	P value
Demographics			
Age (years), mean±SD	70.0±6.2	68.9±5.5	0.176
Gender, n (%)			0.438
Female	32 (29.1)	38 (33.9)	
Male	78 (70.9)	74 (66.1)	
Marital status, n (%)			0.115
Married	76 (69.1)	66 (58.9)	
Single/divorced/widowed	34 (30.9)	46 (41.1)	
Employment status, n (%)			-
Employed	0 (0.0)	0 (0.0)	
Unemployed	110 (100.0)	112 (100.0)	
Education level, n (%)			0.812
Primary school or below	21 (19.1)	18 (16.1)	
Middle or high school	59 (53.6)	64 (57.1)	
Undergraduate or above	30 (27.3)	30 (26.8)	
Location, n (%)			0.951
Urban	91 (82.7)	93 (83.0)	
Rural	19 (17.3)	19 (17.0)	
Smoke history, n (%)			0.579
No	63 (57.3)	60 (53.6)	
Yes	47 (42.7)	52 (46.4)	
Medical histories			
Hypertension, n (%)			0.586
No	60 (54.5)	57 (50.9)	
Yes	50 (45.5)	55 (49.1)	
Hyperlipidemia, n (%)			0.815
No	78 (70.9)	81 (72.3)	
Yes	32 (29.1)	31 (27.7)	
Diabetes, n (%)			0.057
No	82 (74.5)	95 (84.8)	
Yes	28 (25.5)	17 (15.2)	
Disease information			
Diagnosis, n (%)			0.680
Gastric cancer	50 (45.5)	54 (48.2)	
Colorectal cancer	60 (54.5)	58 (51.8)	
ECOG PS score, n (%)			0.253
0	53 (48.2)	60 (53.6)	
1	49 (44.5)	50 (44.6)	
2	8 (7.3)	2 (1.8)	
Differentiation, n (%)			0.828
Well	12 (10.9)	9 (8.0)	
Moderate	45 (40.9)	49 (43.8)	
Poor	53 (48.2)	54 (48.2)	
Multiple metastases, n (%)			0.410
No	64 (58.2)	59 (52.7)	
Yes	46 (41.8)	53 (47.3)	
Lung metastasis, n (%)			0.698
No	61 (55.5)	65 (58.0)	
Yes	49 (44.5)	47 (42.0)	
Liver metastasis, n (%)			0.892
No	56 (50.9)	56 (50.0)	
Yes	54 (49.1)	56 (50.0)	
Peritoneum metastasis, n (%)			0.333
No	74 (67.3)	82 (73.2)	
Yes	36 (32.7)	30 (26.8)	
Other metastases, n (%)			0.058
No	80 (72.7)	68 (60.7)	
Yes	30 (27.3)	44 (39.3)	
Treatment information			
Chemotherapy, n (%)			0.505
No	15 (13.6)	12 (10.7)	
Yes	95 (86.4)	100 (89.3)	
Anti-angiogenesis agent, n (%)			0.134
No	65 (59.1)	77 (68.8)	
Yes	45 (40.9)	35 (31.3)	
Targeted agent, n (%)			0.978
No	66 (60.0)	67 (59.8)	
Yes	44 (40.0)	45 (40.2)	
Immunotherapy, n (%)			0.388
No	81 (73.6)	88 (78.6)	
Yes	29 (26.4)	24 (21.4)	
Assessment at M0, mean±SD			
HADS-A score	7.8±2.4	7.9±2.6	0.746
HADS-D score	7.7±2.6	7.9±2.7	0.752
FACIT-Sp score	32.6±4.9	32.4±5.1	0.778
QLQ-C30 global health status score	59.8±13.8	59.4±14.0	0.835
QLQ-C30 functions score	58.6±15.0	57.9±14.7	0.716
QLQ-C30 symptoms score	37.6±14.8	38.3±16.8	0.751

SD: standard deviation; ECOG PS: Eastern Cooperative Oncology Group Performance Status; M0: at baseline; HADS-A, Hospital Anxiety and Depression Scale-anxiety; HADS-D: Hospital Anxiety and Depression Scale-depression; FACIT-Sp: the Functional Assessment of Chronic Illness Therapy-Spiritual Well-Being Scale; QLQ-C30: Quality of Life Questionnaire-Core 30. *t*-test or chi-squared test.

### Effect of RT on anxiety (primary outcome)

**Figure 2:**
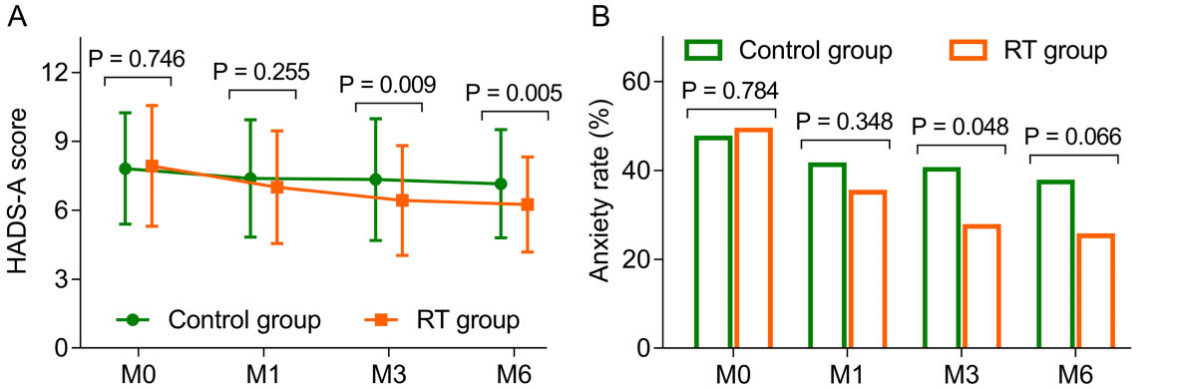
Addition of reminiscence therapy (RT) decreased anxiety in elderly patients with unresectable, metastatic gastrointestinal cancer. Comparison of Hospital Anxiety and Depression Scale for Anxiety (HADS-A) scores (**A**) and anxiety rates (**B**) between the RT group and control group at 0, 1, 3, and 6 months (M). Data are reported as means±SD (*t*-test and chi-squared test).

HADS-A scores at M3 (6.4±2.4 *vs* 7.3±2.6, P=0.009) and M6 (6.3±2.1 *vs* 7.2±2.4, P=0.005) were lower in the RT group than in the control group, while no difference in HADS-A scores at M0 (7.9±2.6 *vs* 7.8±2.4, P=0.746) or M1 (7.0±2.5 *vs* 7.4±2.5, P=0.255) was found between the two groups (Figure 2A). The anxiety rate at M3 (27.4 *vs* 40.2%, P=0.048) decreased in the RT group compared with the control group, and the rate at M6 (25.3 *vs* 37.4%, P=0.066) showed a decreasing trend (without statistical significance) in the RT group compared with the control group. However, the anxiety rates at M0 (49.1 *vs* 47.3%, P=0.784) and M1 (35.1 *vs* 41.3%, P=0.348) did not vary between the two groups (Figure 2B).

For the primary outcome, a multivariate linear regression model showed that the RT group was independently associated with a reduced HADS-A score at M6 (P=0.003), while worse tumor differentiation was independently associated with an increased HADS-A score at M6 (P=0.008) (Supplementary Table S1).

### Effect of RT on depression (secondary outcome)

**Figure 3:**
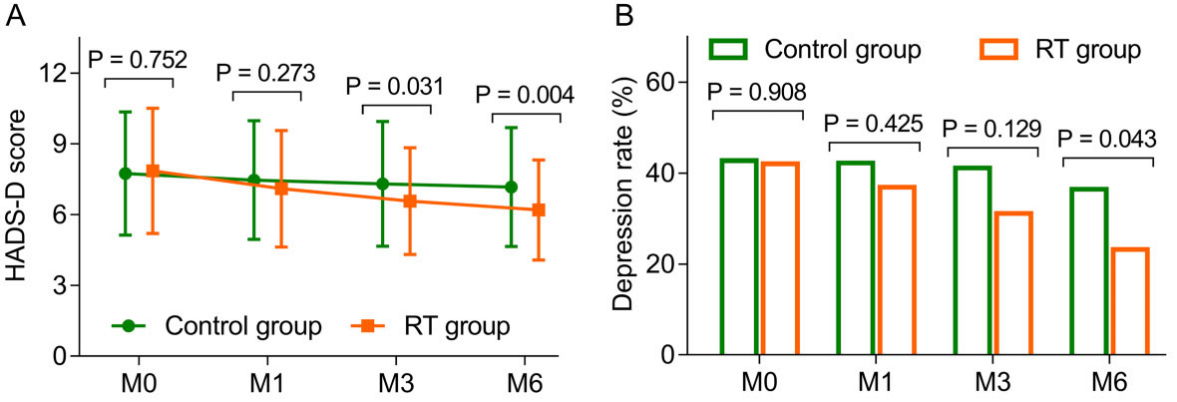
Addition of reminiscence therapy (RT) decreased depression in elderly patients with unresectable, metastatic gastrointestinal cancer. Comparison of Hospital Anxiety and Depression Scale for Depression (HADS-D) scores (**A**) and depression rates (**B**) between the RT group and control group at 0, 1, 3, and 6 months (M). Data are reported as means±SD (*t*-test and chi-squared test).

The HADS-D scores at M3 (6.6±2.3 *vs* 7.3±2.6, P=0.031) and M6 (6.2±2.1 *vs* 7.2±2.5, P=0.004) were lower in the RT group than in the control group, while the HADS-D scores at M0 (7.9±2.7 *vs* 7.7±2.6, P=0.752) and M1 (7.1±2.5 *vs* 7.5±2.5, P=0.273) were not significantly different between the two groups (Figure 3A). The depression rate at M6 (23.2 *vs* 36.4%, P=0.043) was lower in the RT group than in the control group, while the rates at M0 (42.0 *vs* 42.7%, P=0.908), M1 (36.9 *vs* 42.2%, P=0.425), and M3 (31.1 *vs* 41.1%, P=0.129) were not significantly different between the two groups (Figure 3B).

### Effect of RT on spiritual well-being (secondary outcome)

**Figure 4:**
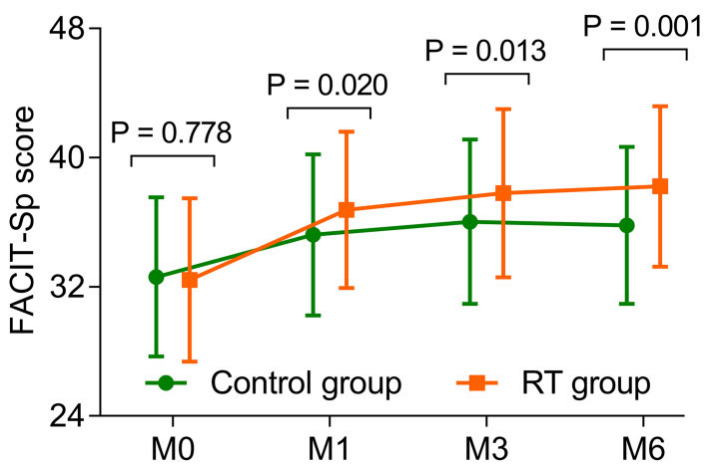
Addition of reminiscence therapy (RT) elevated spiritual well-being in elderly patients with unresectable, metastatic gastrointestinal cancer measured by the Functional Assessment of Chronic Illness Therapy-Spiritual Well-Being Scale (FAVIT-Sp) at 0, 1, 3, and 6 months (M). Data are reported as means±SD (*t*-test).

The FACIT-Sp scores at M1 (36.8±4.8 *vs* 35.2±5.0, P=0.020), M3 (37.8±5.2 *vs* 36.0±5.1, P=0.013), and M6 (38.2±5.0 *vs* 35.8±4.9, P=0.001) were elevated in the RT group compared to the control group. However, the FACIT-Sp score at M0 (32.4±5.1 *vs* 32.6±4.9, P=0.778) was not different between the RT group and the control group (Figure 4).

### Effect of RT on QoL (secondary outcome)

**Figure 5:**
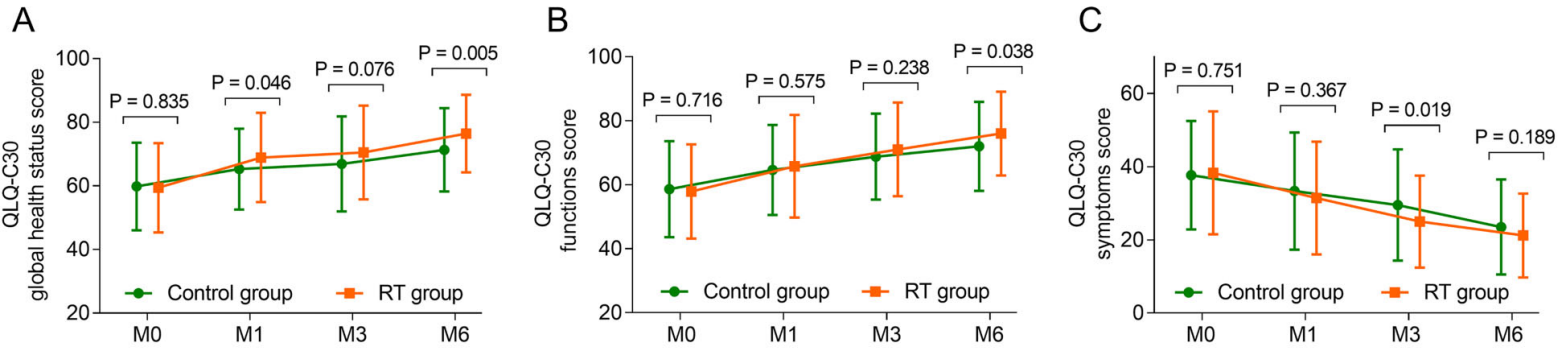
Addition of reminiscence therapy (RT) elevated the quality of life (QoL) in elderly patients with unresectable, metastatic gastrointestinal cancer. Comparison of the Quality of Life Questionnaire-Core 30 (QLQ-C30) global health status score (**A**), QLQ-C30 functions score (**B**), and QLQ-C30 symptoms score (**C**) between the reminiscence therapy (RT) group and control group at 0, 1, 3, and 6 months (M). Data are reported as means±SD (*t*-test).

Similarly, compared with those in the control group, the QLQ-C30 global health status scores at M1 (68.9±14.0 *vs* 65.3±12.7, P=0.046) and M6 (76.4±12.2 *vs* 71.3±13.1, P=0.005) were elevated in the RT group, while the scores at M0 (59.4±14.0 *vs* 59.8±13.8, P=0.835) and M3 (70.5±14.7 *vs* 66.9±14.9, P=0.076) did not vary between the two groups (Figure 5A).

Similarly, the QLQ-C30 score at M6 (76.0±13.1 *vs* 72.0±13.9, P=0.038) was greater in the RT group than in the control group, while the scores at M0 (57.9±14.7 *vs* 58.6±15.0, P=0.716), M1 (65.8±16.0 *vs* 64.7±14.1, P=0.575), and M3 (71.1±14.6 *vs* 68.8±13.4, P=0.238) were not different between the two groups (Figure 5B).

The RT group had lower QLQ-C30 symptoms scores at M3 (25.0±12.6 *vs* 29.5±15.2, P=0.019) than did the control group, but the scores at M0 (38.3±16.8 *vs* 37.6±14.8, P=0.751), M1 (31.4±15.4 *vs* 33.3±16.0, P=0.367), and M6 (21.2±11.5 *vs* 23.5±13.0, P=0.189) were not significantly different between the two groups (Figure 5C).

### Subgroup analysis

In patients with no anxiety at M0, only the FACIT-Sp score at M6 (P=0.029) was increased in the RT group compared with the control group. In patients with anxiety at M0, the HADS-A score (P=0.003) and HADS-D score (P=0.001) at M6 were lower, but the FACIT-Sp score (P=0.009) and QLQ-C30 global health status score (P=0.001) at M6 were greater in the RT group than in the control group.

Among patients with no depression at M0, HADS-A score, HADS-D score, FACIT-Sp score, and QLQ-C30 score at M6 were not significantly different between the RT group and the control group (all P>0.050). In patients with depression at M0, the HADS-A score (P=0.040) and HADS-D score (P<0.001) were lower, whereas the FACIT-Sp score (P<0.001), QLQ-C30 global health status score (P=0.005), and QLQ-C30 function score (P=0.037) at M6 were greater in the RT group than in the control group (Table 2).

**Table 2:** Subgroup analysis of HADS-A, HADS-D, FACIT-Sp, and QLQ-C30 scores at M6 between control group and RT group of elderly patients with unresectable, metastatic gastrointestinal cancer treated with regular therapy (control) or with regular therapy + reminiscence therapy (RT).

Items	Control group	RT group	P value
No anxiety at M0, mean±SD			
HADS-A score	6.4±2.1	5.9±2.0	0.236
HADS-D score	6.7±2.5	6.4±2.0	0.512
FACIT-Sp score	35.2±4.6	37.4±5.2	0.029
QLQ-C30 global health status score	74.5±12.4	76.5±12.0	0.406
QLQ-C30 functions score	72.3±12.7	76.6±13.0	0.088
QLQ-C30 symptoms score	22.1±13.0	20.7±10.1	0.558
Anxiety at M0, mean±SD			
HADS-A score	8.0±2.3	6.6±2.1	0.003
HADS-D score	7.7±2.5	6.0±2.3	0.001
FACIT-Sp score	36.4±5.1	39.1±4.7	0.009
QLQ-C30 global health status score	67.8±13.1	76.3±12.5	0.001
QLQ-C30 functions score	71.7±15.3	75.4±13.3	0.214
QLQ-C30 symptoms score	25.0±13.0	21.7±12.8	0.204
No depression at M0, mean±SD			
HADS-A score	7.1±2.3	6.3±2.2	0.053
HADS-D score	6.1±2.1	5.9±2.0	0.651
FACIT-Sp score	36.6±4.2	37.7±5.0	0.236
QLQ-C30 global health status score	75.1±11.4	77.6±11.1	0.250
QLQ-C30 functions score	74.1±12.9	76.1±13.1	0.398
QLQ-C30 symptoms score	21.0±12.2	20.9±12.3	0.970
Depression at M0, mean±SD			
HADS-A score	7.2±2.4	6.2±1.9	0.040
HADS-D score	8.6±2.3	6.7±2.2	<0.001
FACIT-Sp score	34.7±5.4	39.0±5.0	<0.001
QLQ-C30 global health status score	66.3±13.5	74.8±13.5	0.005
QLQ-C30 functions score	69.3±14.9	75.8±13.2	0.037
QLQ-C30 symptoms score	26.7±13.4	21.6±10.4	0.054

Data are reported as mean±SD. Student’s *t*-test. SD: standard deviation; HADS-A: Hospital Anxiety and Depression Scale-anxiety; HADS-D: Hospital Anxiety and Depression Scale-depression; FACIT-Sp: Functional Assessment of Chronic Illness Therapy-Spiritual Well-Being Scale; QLQ-C30: Quality of Life Questionnaire-Core 30.

## Discussion

This study in elderly patients with unresectable, metastatic GI cancer revealed the following findings: 1) The addition of RT reduced anxiety and depression; 2) Spiritual well-being was improved by the addition of RT; and 3) The addition of RT improved the QoL (concerning global health status, function, and symptoms).

The emergence of RT provides a novel option for anxiety and depression management in GI cancer patients (9,14). For instance, a previous study revealed that RT decreases anxiety but has a relatively low effect on relieving depression in postoperative gastric cancer patients (9). Another study revealed that RT intervention is effective at reducing the symptoms of anxiety and depression in patients with digestive system cancer (14). The possible reasons for the present findings might be as follows: 1) Cancer patients typically experience loneliness, while RT provides them with an opportunity to share memories with others, ultimately alleviating feelings of isolation and loneliness (15); 2) RT might be involved in the regulation of neurotransmitters, including dopamine, 5-hydroxytryptamine, and norepinephrine, thus attenuating psychological disorders (16).

Spiritual well-being refers to positive engagement with others, the self, and the environment and is commonly measured by the FACIT-Sp (11,17,18). However, limited studies have reported the influence of RT on spiritual well-being in elderly patients with unresectable, metastatic GI cancer. The explanation for our findings might be that RT facilitates communication and fosters the self-identity of patients, ultimately improving their quality of life (19). Moreover, this study revealed that the addition of RT improved FACIT-Sp scores at the early phase of intervention (M1), while HADS-A and HADS-D scores did not vary until M3. This contrast indicated that the addition of RT was more effective at improving spiritual well-being. Nonetheless, further studies are needed to validate this issue.

Improving QoL is essential throughout GI cancer management because worse QoL is associated with shorter overall survival (20). Recent studies suggest that RT is effective at improving QoL among cancer patients (16,21,22). For example, one study revealed that RT exhibits satisfactory efficacy in elevating the global health status and function dimension of the QLQ-C30 in colorectal cancer patients (22). Another study revealed that RT improves QoL in patients with advanced cancer, including GI cancer (21). The possible reasons for our findings might be as follows: 1) As mentioned above, RT relieves anxiety and depression and improves spiritual well-being, which increases patients' compliance with cancer-related treatment and thus ameliorates common symptoms of GI cancer, including fatigue, dry mouth, and pain (23); 2) Recalling of past events during RT encourages patients to focus on positive experiences, thus fostering a more optimistic attitude toward life (22,24). Furthermore, this study revealed that the addition of RT noticeably reduced anxiety and depression and increased spiritual well-being and QoL in patients with anxiety or depression at M0 compared to those without. A possible reason for this result might be that patients with anxiety or depression at M0 encounter worse psychological conditions, and the treatment benefit is higher after receiving RT intervention (25).

There were several limitations in the present study. First, this was a single-center study; thus, selection bias was difficult to avoid. Second, a six-month follow-up duration was used to assess the influence of RT on anxiety, depression, spiritual well-being, and QoL, but the effect of RT in the longer term requires further research. Finally, this study used only the HADS to assess anxiety and depression status, thus additional alternative scales should be applied to validate the findings.

Above all, addition of RT is not only a potential modality to relieve anxiety and depression measured by HADS, but also a supportive therapy to improve spiritual well-being assessed using FACIT-Sp and QoL in dimensions of global health, functions, and symptoms evaluated by QLQ-C30 within 6 months in elderly patients with unresectable, metastatic GI cancer.

## Supplementary Material

Click here to view [pdf].
