# Supplementary material for: Influence of reminiscence therapy on mental health and quality of life in elderly patients with unresectable, metastatic gastrointestinal cancer
Source: Braz J Med Biol Res. 2024 May 20;57:e13344. doi: 10.1590/1414-431X2024e13344 (PMC11136486; doi:10.1590/1414-431X2024e13344)
Supplement: Supplementary file 1 [file 1414-431X-bjmbr-57-e13344-suppl.pdf]

Table S1. Multivariate linear regression model for HADS-A at M6 of elderly patients with unresectable, metastatic gastrointestinal cancer treated with regular therapy (control) or with regular therapy + reminiscence therapy (RT).

Items	Unstandardized Coefficient		Standardized Coefficient	*t*	P
	B	SE	β		
Intervention, RT group *vs* control group	–0.930	0.311	–0.206	–2.993	0.003
Worse differentiation	0.623	0.234	0.183	2.659	0.008

HADS-A: Hospital Anxiety and Depression Scale-anxiety; SE: standard error.
